# The impact of differential pricing subject on consumer behavior

**DOI:** 10.1186/s40359-024-01928-x

**Published:** 2024-08-09

**Authors:** Jinsong Chen, Yuexin Zhang, Yumin Wu

**Affiliations:** 1 School of Business Administration, Guizhou University of Finance and Economics, Guiyang, Guizhou 550025 The People’s Republic of China; 2 School of Culture and Tourism, Chongqing City Management College, Chongqing, 401331 The People’s Republic of China

**Keywords:** Artificial intelligence, Pricing, Mind perception, Ethical perception, Perceived enterprise control, Consumer behavior

## Abstract

The escalating use of artificial intelligence in marketing significantly impacts all aspects of consumer life. This research, grounded in attribution theory and S–O-R theory, employs scenario-based experimental methods to simulate two distinct purchasing contexts. The aim is to investigate consumers' psychological and behavioral responses to AI-initiated pricing. Through SPSS analysis of variance and Bootstrap analysis, the mechanisms of influence of AI-initiated pricing on consumer behavior are tested, revealing the mediating variables of mind perception and consumer perceived ethicality, as well as the mediating variables of perceived enterprise control. Data were collected from Chinese customers to test the model of this study. A total of 841 valid questionnaires were analyzed using ANOVA and Bootstrap analysis with SPSS. The results show that: (1) Consumers exhibit higher repurchase and word-of-mouth recommendation behaviors and lower complaint and switching behaviors for AI-initiated pricing compared to marketers; (2) AI-initiated pricing leads to diminished mind perceptions and augmented ethical perceptions among consumers. Ethical perceptions serve as a complete mediator, while mind perceptions play a less significant mediating role; (3) Perceived enterprise control plays a moderating role in the impact of AI-initiated pricing on consumer behavior. That is, when consumers know that the enterprise can control pricing agents, AI-initiated pricing leads to lower repurchase and word-of-mouth recommendation behaviors, and higher instances of complaining and switching behaviors than humans.

## Introduction

### Background

Leveraging AI for pricing products and services is emerging as a prevalent trend. AI possesses the capability to dynamically adjust prices and utilize consumers' personal data (such as purchase history, geographic location, and product preferences) to forecast purchasing intentions and apply personalized pricing strategies [29]. While this approach can maximize consumer surplus for businesses, it also raises significant ethical concerns and potential negative ramifications for enterprises. For instance, consumers often perceive it as unfair when they discover they are charged more than others for the same product [18]. This perception can lead to negative emotions [14, 63, 64], diminish trust in the merchant or platform [18], and decrease their purchasing intent and propensity to seek alternatives [19]. Furthermore, they may engage in self-protective actions like spreading negative word-of-mouth or lodging complaints [63] This presents a quandary for businesses: while AI algorithms can enhance profitability, they also risk inciting consumer dissatisfaction and adverse reactions. Thus, the ethical and moral considerations of such varied pricing practices warrant thorough examination.

### Research gap

Campbell [8] introduced the "Effect of Source" theory, positing that the origins of information regarding price increases impact consumer's motivational inferences and their assessment of price fairness. This theory suggests a differentiated perception of fairness when prices are communicated by human agents (e.g., salespersons) compared to inanimate sources (e.g., price tags) [8]. The price source effect may indeed be attributed to AI's unique characteristics as an "inanimate object," capable of rapidly adjusting and setting product prices. This aspect of AI, along with its efficiency and precision in price management, introduces a novel dynamic to consumer interactions with pricing strategies.

Historically, the impact of different price-setters on consumer behavior has been underexplored, marking a significant research gap. This gap is particularly evident in the exploration of how varying pricing agents (AI versus marketers) influence consumer behavior. Moreover, while existing research predominantly focuses on the implications of price fairness on consumer psychology and behavior [23, 63], the role of AI in pricing strategies introduces complex ethical considerations [51]. The academic community remains divided on whether AI should be considered an ethical agent accountable for its actions. This division prompts a critical examination of AI's role in ethical decision-making and its potential responsibilities.

### Research contribution

In response to these considerations, this paper aims to bridge the research gap by analyzing the impact of different pricing subjects (AI vs. marketers) on consumer behavior from ethical and mind perspectives. The contributions of this paper are fivefold. First, it quantifies the varying impacts of AI-initiated vs. marketer-initiated differential pricing on consumer behaviors, including repurchase, word-of-mouth recommendation, complaints, and switching, enhancing our understanding of human-AI contrasts in marketing and expanding research on algorithmic pricing.

Second, this study applies attribution and S–O-R theories to explore the mediating effects of pricing subjects (AI vs. marketers) on consumer responses, offering new insights into the psychological impacts of price variations. This clarifies AI-initiated pricing's influence on consumer psychology and behavior, enriching the literature on mind and ethical perceptions in pricing.

Third, the research delineates the boundary conditions for enterprise control's impact on consumer reactions to differential pricing by AI and marketers, exploring how control perceptions modulate these effects.

Fourth, it can assist relevant companies and platforms in gaining a deeper understanding of consumers' psychological perceptions and behavioral responses to AI pricing. It can also provide valuable insights for future marketing communications or service improvements.

Fifth, this study will highlight to companies and platforms that AI pricing is not a one-size-fits-all solution and does not come without potential drawbacks. The findings of this study will enable enterprises and platforms to comprehend the dual aspects of AI pricing, aiding them in formulating balanced pricing strategies.

## Conceptual framework and hypotheses development

### Concepts

#### Differentiated pricing subjects and consumer correspondence

Differentiated pricing in e-commerce can be classified as dynamic pricing and personalized pricing. These forms are based on factors such as purchase history, time, geographic location, and facial recognition. All these types of differentiated pricing have been shown to negatively impact consumers to varying degrees [23, 36, 45, 57]. Previous studies have primarily focused on the negative impact on price-disadvantaged consumers. However, differentiated pricing does not necessarily benefit price-advantaged consumers. For example, Hufnagel et al. [29] found that price-advantaged consumers still perceive lower price fairness due to guilt, resulting in lower purchase intention [29]. Additionally, consumers with strong social norms criticize differentiated pricing even when they benefit from price differences [2].

Consumers' negative perceptions of dynamic pricing vary depending on buyer characteristics. Consumers find online retailers who offer lower prices to new customers, while identifying existing customers, less trustworthy than those who use purchase time as a price determinant [61]. However, some studies have found that dynamic pricing does not significantly affect consumer trust [33]. To assess consumer behavior, studies commonly measure willingness to buy, switch, complain, and search for alternatives in response to differentiated pricing. Differential pricing generally reduces consumers' willingness to buy [19, 29] and to recommend by word-of-mouth [34], while increasing their willingness to complain (both privately and publicly) and to switch [34, 43].

#### AI-initiated pricing

Artificial Intelligence initiate pricing is essentially algorithmic pricing (AIP). Algorithmic pricing uses data analytics to calculate prices based on various parameters at lightning speed, generating dynamic prices in real time [49]. There are two forms of algorithmic pricing: dynamic pricing and personalized pricing. Dynamic pricing adjusts prices dynamically to achieve revenue gains in uncertain market conditions. Personalized pricing, or first-degree price discrimination, charges different prices to different consumers based on their willingness to buy. This form of price differentiation leverages varying prices for the same product based on different customers. It's an important strategy for firms to profit from the heterogeneity in consumer tastes and valuations [43]. In the online environment, collecting necessary customer information is straightforward [19]. Customer data can now be combined with machine learning and optimization tools to predict an individual's willingness to pay and set prices accordingly.

#### Mind perception

Mind perception (MP) denotes an individual's capacity to infer the mental states, objectives, beliefs, and emotions of a physical object by postulating unobservable attributes, which act as mediators between sensory inputs and subsequent behaviors [21, 22]. Firstly, mind perception is a psychological factor that influences consumers' views of AI products or services. People tend to be more receptive to AI handling tasks that are dynamic in nature, whereas they exhibit resistance or aversion towards AI in roles that demand sensibility [58]. AI entities that exhibit more human-like traits are more likely to be attributed with mental states by consumers [30], being perceived as more empathetic [37]. Consequently, enhancing the anthropomorphic features of AI can amplify consumer mind perception, thereby increasing their acceptance of AI products and services [46].

However, it has been observed that in instances of AI service failures, a heightened mind perception correlates with stronger consumer responsibility attribution towards the AI [27]. Secondly, mind perception serves as a crucial differentiator between human and AI competencies [35] Robots are often perceived as less insightful and less dynamic compared to humans [37], which means algorithmic errors tend to trigger a lesser brand crisis than similar errors made by humans [52].

***Ethical perception.***

Judgments of morality form the basis of individual decision-making. They inform personal moral behavior and influence interpersonal and social interactions [9]. People prefer to associate with those they perceive as ethical and avoid those they do not. In marketing, Consumer Perceived Ethicality (CPE) refers to a consumer's ethical judgment about a subject (e.g., a company, brand, product, or service), representing their overall subjective impression of morality [5].

Consumer ethical perception involves three groups: those disadvantaged by price differentials, those benefiting from them but critical of the practice [57], and those unaffected but who ethically question differential pricing [2]. Consumers disadvantaged by price differentiation often feel strong immorality and unfairness. Perceptions of a firm's ethical failure, especially regarding price, can significantly alter consumer relationships with the firm [31, 53] or lead to negative, even punitive responses. This undoubtedly damages trust between consumers and firms [16], indirectly harming brand loyalty and reducing long-term profitability [26].

### Theoretical framework

#### Attribution theory

Attribution theory refers to the process whereby individuals analyze behaviors, attributing causes to actors or external environments [60]. Weiner [59] delineated that attributions for events or behaviors should be assessed across three dimensions: locus of causality (factor source), controllability, and stability [59]. Previous research has confirmed attribution theory's role in understanding how price increases affect consumer behavior. Initially, consumers attribute the source of price increases. Consumers perceive price increases aimed at boosting merchant profits as unfair [6, 7]. Conversely, they show understanding when increases result from external factors, such as government-mandated price adjustments [64].

Furthermore, consumers will closely examine the specific merchant factors contributing to the price increase. The source effect indicates that when consumers learn a price increase stems from the merchant, not the product itself, their perception of price fairness diminishes [6]. He [25] discovered consumers perceive AI-generated price differences as fairer than those set by business personnel. Additionally, consumers consider the controllability of price increases. Consumers are less willing to purchase if the business can control the cause of the price increase [55, 64]. For instance, price hikes due to inflation are seen as uncontrollable, whereas those initiated by supermarkets lead to reduced purchase intentions. Zhang and Zhong [65] identified an interaction between controllability and the source of product price increases [65].

Lastly, Ding [14] observed consumers view temporary price increases as more unfair than permanent ones [14]. Haws & Bearden [23] discovered that consumers are particularly sensitive to price discrepancies between themselves and others. When realizing they are paying more than others for the same product, consumers typically feel a sense of injustice and experience negative emotions [23].

Attribution theory is crucial in explaining consumer behavior and influencing responses to service failures or price hikes across its dimensions. This study employs attribution theory to explore how different sources (AI vs. marketers) influence consumer responses to price disadvantages, alongside exploring the moderating role of controllability (perceived enterprise control).

#### SOR theory

SOR theory (Stimulus-Organism-Response) suggests that stimuli, as extrinsic influences, affect people's mental states, prompting responses [42]. After a series of psychological processes, receivers exhibit intrinsic or extrinsic behavioral responses. Intrinsic responses involve individual attitudes, while behavioral responses include approach or avoidance behaviors [16].

John B. Watson, the founder of the S–O-R theory, divided individual behavior into stimulus and response. While the S-R model elucidates the causal relationship of individual behaviors, it overlooks the individual's internal processes. To explore the "black box" of an individual's internal activities, Mehrabian [39] enhanced the S-R model by introducing the mediating variable "organism," leading to the "Stimulus-Organism-Response" model [39]. The term "organism" describes the internal cognitive and affective processes that an individual undergoes between receiving a stimulus and exhibiting behavior, encompassing both cognition and emotion [16]. The cognitive state involves the mental processes of acquiring, processing, retaining, and retrieving information, while the affective state pertains to the emotions consumers exhibit in response to stimuli [39]. Afterward, the S–O-R model was applied in the field of marketing, elucidating the general process of consumer purchase decisions. Response refers to the final action or attitude of consumers, which can be categorized as positive reactions or negative behaviors [39].

In marketing research, consumer purchase intention is commonly employed as an outcome variable within the S–O-R framework [10, 44]. In the context of a service environment, Bitner [3] observed that external stimuli elicit cognitive, affective, and physical responses from consumers, collectively influencing their actual behavior [3]. The shopping environment, serving as the primary stimulus, shapes consumer behavior through the store's ambiance, influenced by color schemes, lighting, music, and scent, which engage the consumers' visual, auditory, and olfactory senses [16, 39]. Consequently, this study explores consumer behavioral responses to varied pricing strategies (AI versus marketers) as stimuli, focusing on mental and ethical perceptions as intermediary processes.

Widely applied in consumer behavior research, SOR theory explains how events shape consumer perceptions and subsequent behaviors. This study examines organisms from cognitive and affective perspectives and consumer reactions through purchase intentions. Previous researches show diverse personnel characteristics influence consumer psychology and behavior [28, 40], yet AI's role as a stimulus in consumer behavior remains underexplored. Hence, this study investigates how consumers respond to differentiated pricing stimuli (AI vs. marketers) and their mental and ethical perceptions.

**Fig. 1:**
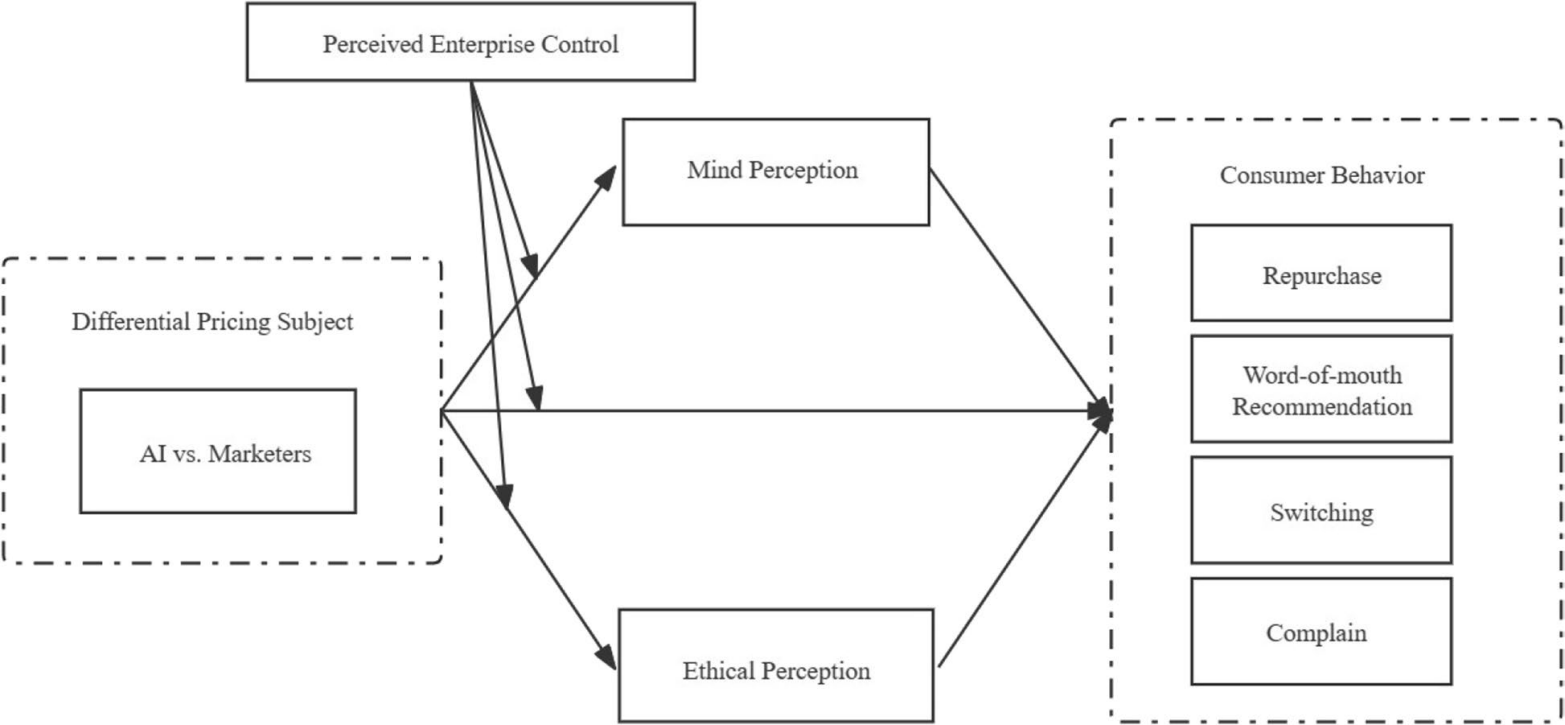
Conceptual model

The research model is shown in Fig. 1.

### Hypotheses development

#### Differentiated pricing strategies (AI vs marketer) and consumer behavior

When consumers realize they are paying a higher price than others for the same product, they often feel it is unfair, leading to negative emotions such as anger and loneliness. This reduces their trust in the merchant or platform, decreases their willingness to buy and search for products, and may even lead to self-protective behaviors like spreading negative word-of-mouth and filing complaints.

Additionally, consumers analyze the reasons behind these price differences and exhibit varying behavioral responses based on their analytical judgments. Attribution theory posits individuals attribute events or behaviors from three perspectives: factor source, controllability, and stability. In scenarios involving service failures or price disparities, consumers first assess the source of the factor: internal attribution (reasons related to the consumer) and external attribution (attributed to other agents or environmental factors). Generally, consumers tend to make external attributions, inferring motives or intentions from observed behaviors and outcomes.

When identifying the source of a service failure, consumers are more inclined to consider a "person" rather than an inanimate object (like AI or a machine). On one hand, AI, as an inanimate entity, is often perceived by consumers as a mechanical or coded entity lacking the consciousness of living beings, leading to a distinction between AI and humans. On the other hand, consumers tend to have stronger emotional responses to humans. Positive outcomes are more likely attributed to human intervention than to machines [47], and conversely, failures are also more often blamed on human error rather than on machines [41].

Therefore, when faced with price differences, consumers disadvantaged by pricing are more likely to attribute these disparities to marketers, linking the difference more to the firm or platform, and as a result, may exhibit lower repurchase and word-of-mouth referral behaviors, and higher instances of complaining and switching behaviors. Based on this, we propose H1, H1a, H1b, H1c, H1d.

- H1: Consumers have a positive reaction to AI-initiated pricing than to marketer-initiated.
- H1a: AI-initiated pricing leads to more consumer repurchase behavior than marketers.
- H1b: AI-initiated pricing leads to more consumer word-of-mouth recommendation behavior than marketers.
- H1c: AI-initiated pricing leads to less complaint behavior than marketers.
- H1d: AI-initiated pricing leads to less switching behavior than marketers.

#### Mediator role of mind perception

Artificial intelligence is a neutral technology. Consumers typically view it as an inanimate machine or code, lacking thoughts, emotions, and ethical or moral consciousness. Consequently, they perceive AI as having a lower level of agency than humans [52]. Therefore, this paper argues that consumers have a lower mind perception of AI compared to marketers.

Moreover, an individual's perception of an entity's intentions and behaviors influences their attribution of responsibility for the entity's actions, leading to varied evaluations and reactions. When consumers recognize that a pricing discrepancy is determined by artificial intelligence, they tend to have a lower mind perception of these intelligent pricing algorithms, as they lack "human-like" characteristics [38]. This may lead consumers to view the price difference as a result of an algorithmic system error or a random occurrence, devoid of subjective intention, and not as a deliberate act by the company.

In contrast, when consumers understand that the price difference is set by human marketers, they may perceive it as a purposeful act by the marketers to achieve their personal objectives or to advance the firm's profit goals. Consequently, this understanding can prompt more negative behavioral responses from consumers. So, we propose H2 and H3.

- H2: Consumers have lower mind perception towards AI-initiated pricing than marketers.
- H3: Mind perception mediates differentiated pricing subjects (AI vs. marketer) and consumer behavior. That is, Consumers have lower mind perceptions of AI (vs. marketers), which in turn generates higher repurchase and word-of-mouth recommendations and lower switching and complaint behavior.

#### Mediator role of ethical perception

Individuals' ethical decisions are informed by their ethical perceptions and attributions of intent [1]. In attributing intentions, different agents evoke varied ethical responses, with a general trend of greater leniency towards those perceived to have lower ethical agency. Thus, in line with this study's focus, it is posited that consumers attribute higher ethical perception to AI-initiated pricing over marketer-initiated pricing. The perception of ethical shortcomings in a firm significantly influences consumer-firm relationships, potentially diminishing purchase intentions or provoking retaliatory actions [55].

Differentiated pricing, perceived as an infringement of social norms and ethical standards, often elicits consumer disdain, manifesting in reduced purchase likelihood, increased complaints, and switching behaviors. Unlike AI, which is regarded merely as a tool devoid of ethical sensibility, marketers, being capable of empathy and ethical reasoning, are expected to conform to established pricing norms. Consequently, this study argues that consumers attribute a higher ethical perception to AI pricing than to marketer-initiated pricing, resulting in a preference for AI pricing (vs. marketer pricing). Based on this, hypotheses H4a and H4b are proposed.

- H4: Consumers have higher ethical perception towards AI-initiated pricing than marketers.
- H5: Mind perception mediates differentiated pricing subjects (AI vs. marketer) and consumer behavior. That is, consumers have higher ethical perceptions of AI (vs. marketers), which in turn generates higher repurchase and word-of-mouth recommendations and lower switching and complaint behavior.

## Moderating role of perceived enterprise controls

Perceived enterprise control is the consumer's understanding of an enterprise's (or decision-maker's) authority over an event's occurrence and its results. In instances of service failure, consumers assess not only who is responsible for the failure but also if the failure was within the enterprise's control, that is, whether it could have been averted [35].

In this research, perceived enterprise control concerns the degree to which consumers believe enterprises can manage AI or marketer-based pricing. Controllability, a pivotal element in attribution theory, significantly influences consumer behavior. When a failure is attributed to controllability, it can lead to adverse consumer reactions and even retaliatory inclinations [11].

For instance, a service failure attributed to negligence, such as carelessness in a store, typically provokes stronger consumer resentment towards failures seen as preventable [17], subsequently impacting their willingness to interact with the company. Furthermore, the interaction between controllability attribution and factor sources is significant [48].

Research by Zhang and Zhong [65] indicated that consumers are more amenable to price increases driven by uncontrollable factors, such as a rise in raw material costs (external attribution) than those due to controllable factors like equipment upgrades (internal attribution) [52].

Consequently, this study posits that when consumers are aware that enterprises can manipulate pricing through AI or marketers, they may perceive this as a deliberate exploitation of AI's efficient analytical prowess for differentiated pricing and profit maximization. As a result, consumers' negative behavioral responses to AI (vs. marketer)-initiated pricing may not differ significantly or might even invert, lessening or reversing the negative behavioral trends typically associated with marketer-initiated pricing. Based on this, we proposed H6a.

- H6a: Enterprise control diminishes the impact of AI (vs. marketer) initiated pricing on consumers’ behavior, even showing lower repurchase and word-of-mouth recommendation, and higher complaint and switching behavior.

According to the study by Gray et al. [21], people's mind perception of inanimate objects like robots is significantly lower than that of adult humans [9]. Mind perception of a physical object encompasses two dimensions: affective and cognitive. Enhancing these characteristics in AI can potentially elevate its mind perception. When AI systems exhibit empathetic and understanding traits, consumers are more inclined to accept product recommendations from AI customer service. Similarly, augmenting the AI's cognitive mobility can enhance its social interaction capabilities. However, there is a caveat: when AI becomes excessively human-like, it can evoke negative emotions in people, leading ultimately to a rejection of the AI [62].

This study posits that when consumers perceive that enterprises have the ability to control AI pricing, their mind perception of AI equals or even surpasses that of human marketers. This aligns with Hypothesis 2, suggesting that AI algorithms possess less intentional agency in executing differentiated pricing compared to marketers, as algorithmic systems do not form subjective notions, let alone base pricing decisions on such ideas. When consumers understand that an enterprise is manipulating AI for pricing, they perceive it as the firm, rather than the AI, setting differentiated prices.

Consequently, in such scenarios, consumers' mind perception of the AI, as influenced by the enterprise, is on par with their mind perception of the marketer. This perception shifts the attribution of pricing decisions from the AI to the controlling entity, the enterprise, thereby equating the AI's perceived intentionality with that of human marketers. Based on this, we proposed H6b.

- H6b: Perceived enterprise control diminishes the impact of AI (vs. marketer) initiated pricing on mind perception.

The current debate around AI's responsibility for ethical behavior centers on whether AI can develop subjective intentions and execute actions based on them. For instance, when both an AI and a human present a product or service, consumers often believe that the AI lacks selfish intentions, thereby mitigating certain extreme reactive behaviors [20] However, when an enterprise controls AI, it is akin to the enterprise "imparting" its intentions into the AI, particularly in setting differentiated prices. This can lead consumers to perceive such actions as a deliberate ploy by the enterprise to manipulate AI pricing for increased profits.

- H6c: Perceived enterprise control diminishes the impact of AI (vs. marketer) initiated pricing on ethical perception.

## Experimental design

In this study, data collection was conducted through two experiments designed to validate the hypotheses. Initially, a pre-test was conducted for two primary reasons: firstly, to confirm the effectiveness of the experimental stimulus material manipulation, thereby laying the groundwork for the main experiment. Secondly, the pre-test aimed to ascertain whether consumers' responses to AI pricing and marketer pricing are not significantly different in scenarios where no price differentiation exists.

This step was crucial to ensure that any observed variations in consumer behavior in the differentiation scenario are solely attributable to the AI versus marketer variable. The experimental framework employed a 2 (price differentiation: present or absent) × 2 (pricing subject: AI or marketer) between-group design, providing a structured approach to examining the nuanced impacts of these variables on consumer behavior.

## Empirical analysis

### Pretest

We conducted an experimental, scenario-based online study to validate the logical coherence and relevance of the expressions and context embedded within the experimental materials, ensuring they align with the research requirements. All participants provided informed consent before taking part in this study. Consent was obtained in written form. For written consent, participants were provided with a consent form outlining the study's purpose, procedures, potential risks, and benefits. Participants signed the form before participating in the survey.

The recruitment period for this study commenced on 01 February 2022 and concluded on 01 May 2022. For participants under the age of 18, we obtained consent from a parent or guardian in addition to the assent from the minors. The parent or guardian was provided with a detailed consent form and was required to sign it to document their consent. The pre-test setup followed a 2 (price differentiation: present or absent) × 2 (pricing subject: AI or marketer) between-group design.

#### Experimental material

The experiment was structured into four groups, with experimental materials crafted based on the research by Song and He [51] and Srinivasan & Saria-Abi [52]. To mitigate the influence of external factors like website and brand, a fictitious cell phone brand named HMS was created. The specific scenarios presented to each group were as follows:

- Price Difference × AI Group: Please imagine this scenario: HMS Cellular, a top-tier cell phone brand, utilizes pricing robots to determine its product prices. You purchase a cell phone from the HMS store for $4,999. Later, you discover that another customer, Customer A, bought an identical cell phone from the same store on the same day, but paid a lower price of $4,799. It's noteworthy that both you and Customer A are regular patrons of HMS. For more information, refer to the details provided below.
- Price Difference × Marketer Group: Please imagine this scenario: HMS Cellular, a top-tier cell phone brand, utilizes experienced markers to determine its product prices. You purchase a cell phone from the HMS store for $4,999. Later, you discover that another customer, Customer A, bought an identical cell phone from the same store on the same day, but paid a lower price of $4,799. It's noteworthy that both you and Customer A are regular patrons of HMS. For more information, refer to the details provided below.
- No Price Difference × AI Group: Please imagine this scenario: HMS Cellular, a top-tier cell phone brand, utilizes pricing robots to determine its product prices. You purchase a cell phone from the HMS store for $4,999. Later, you discover that another customer, Customer A, also bought an identical cell phone from the same store on the same day, and they paid the same price of $4,999. It's noteworthy that both you and Customer A are regular patrons of HMS. For more information, refer to the details provided below.
- No Price Difference × Marketer's Group: Please imagine this scenario: HMS Cellular, a top-tier cell phone brand, utilizes experienced markers to determine its product prices. You purchase a cell phone from the HMS store for $4,999. Later, you discover that another customer, Customer A, also bought an identical cell phone from the same store on the same day, and they paid the same price of $4,999. It's noteworthy that both you and Customer A are regular patrons of HMS. For more information, refer to the details provided below.

#### Data collection and description

Participants completed the survey on the platform Credamo (www.credamo.com), where they were randomly assigned to one of the scenarios. For their participation, respondents received a cash payment of $ 0.2. In this experiment, All the participants are from China. 200 valid questionnaires were collected. Under the no-price-difference condition, 100 valid responses were received, comprising 27 males and 73 females. Over 40% of these respondents were aged between 18–25 years, with undergraduates constituting 42% of this group. Additionally, 32 participants were students, making up 32% of the total. In the price difference group, 100 valid questionnaires were retrieved, including 51 males and 49 females. Among these, individuals aged 18–25 years represented 55% of the group. Undergraduates accounted for 68% of the responses, and 34% of the participants were employed in private enterprises.

#### Measurement items

The material examination questions were crafted to assess the comprehensibility and clarity of the experimental materials, taking cues from Hufnagel et al. [29]. The key manipulated variable in this study was the subject of differentiated pricing (AI vs. marketers), with measurement questions inspired by the work of Song and He [51]. Regarding consumer behavior measures, the repurchase component included three items, adapted from Victor et al. [56]. The complaint dimension comprised three items, based on Garbarino & Maxwell [19]. The switching aspect involved two items, following Singh [50], and the word-of-mouth recommendation was measured with two items, derived from Lii & Sy [34].

All items were structured on a 7-point Likert scale, where 1 represented "strongly disagree" and 7 signified "strongly agree". This scaling system facilitated the quantification of participant responses, allowing for a nuanced analysis of their attitudes and perceptions regarding differentiated pricing strategies. (see, Table 1, at the end of the article).

**Table 1:** Measurements

Variable	Items	Reference
Stimulate Material	SM1: The scenario presented above is very easy to understand	Hufnagel et al. [29]
Pricing Subject	PS1: Based on the description of the above scenario, who set the price of the cell phone you purchased?	Song & He [51]
	PS2: Who made the price of your cell phone different from Customer A's?	
Repurchase	RP1: I will continue to buy more from this cell phone store for years to come	Victor et al. [56]
	RP2: I will buy other different products from this cell phone store if I need them	
	RP3: I am willing to buy a cell phone from this store if need them	
Complain	CP1: I will complain to my friends and relatives about the cell phone store's behavior	Garbarino & Maxwell [19]
	CP2: I will complain to the store's customer service about the cell phone store's behavior	
	CP3: I will complain to a third party like consumer association about the cell phone store's behavior	
Switching	CS1: The store's differentiated pricing behavior makes me not want to stay in touch with the store	Singh [50]
	CS2: The store's differentiated pricing behavior makes me inclined to buy similar products from other stores	
Word-to-Mouth	WoM1: I will recommend this store to people around me when they need to buy a cell phone	Lii & Sy [34]
	WoM2: I will tell others about the benefits of this cell phone store	
Mind perception	MP1: AI (or Marketers) can formulate a plan	Srinivasan & Saria-Abi and Gray et al. [21, 52]
	MP2: AI (or Marketers) can recognize things right or wrong	
	MP3: AI (or Marketers) is able to do thinking	
	MP4: AI (or Marketers) can express itself	
Ethical perception	CPE1: I think AI-initiated (or Marketer-initiated) pricing is ethical	Brunk [5]
	CPE2: I think AI-initiated (or Marketer-initiated) pricing abide by ethics	
	CPE3: I think the prices set by AI (or Marketers) are fair	
Perceived Cost	Cost1: HMS cell phone stores invest a lot of money in operating (including employee salaries, equipment investment, etc.)	Bolton & Alba [4]
	Cost2: HMS cell phone stores invest a lot of money in marketing	
Emotional State	While answering the question, I was in the mood to:	Lee & Sternthal [32]
	M1: Happy	
	M2: Excited	
	M3: Joyful	
	M4: Depressed	
	M5: Disappointed	
	M6: Angry	
Perceived Enterprise Control	PCC1: The enterprise is able to manipulate the price-setting process of the AI (or marketers)	Song & He [51]
	PCC2: The enterprise has control over the pricing outcome of the product	
	PCC3: The enterprise enables AI (marketers) to price as per the its requirements	

#### Result

The average score for the material test questions was 5.905, with a standard deviation of 1.44, demonstrating that the experimental material was comprehensible to participants. The Cronbach's alpha values for all scales exceeded the critical threshold of 0.7, demonstrating strong reliability, including repurchase (Cronbach's alpha = 0.915), complaint (Cronbach's alpha = 0.865), switching (Cronbach's alpha = 0.813), word-of-mouth (Cronbach's alpha = 0.935).

Furthermore, regarding the perception of the manipulated variable, the pricing subject, its successful manipulation was examined through one-factor analysis. The results, as shown in Table 2, indicate that in the no price difference group, M_AI_ = 4.58 and M_marketer_ = 2.49, with *p* = 0.000. This significant difference confirmed the successful manipulation of the experimental question items. Similarly, in the price difference group, M_AI_ = 6.02 and M_marketer_ = 1.47, with *p* = 0.000, also indicating a significant difference.

**Table 2:** Manipulation results

Experimental Scenario	Group	N	M	SD	F	p
No Pricing difference	AI	50	4.58	1.55	66.50	0.000
	Marketer	50	2.49	0.94		
Pricing difference	AI	50	6.02	1.48	413.426	0.000
	Marketer	50	1.47	0.55		

Next, consumer responses under the no price difference condition were evaluated using one-factor ANOVA, with results presented in Table 3. There was no significant difference (*p* > 0.05) in repurchase (*p* = 0.295), word-of-mouth recommendation (*p* = 0.549), complaint (*p* = 0.549), and switching (*p *= 0.890), implying consistency and no variation in consumer responses to AI and marketer pricing in terms of repurchase, word-of-mouth, complaint, and switching behaviors.

**Table 3:** Results of differentiated pricing subject on consumer behavior in scenarios without price differences

Variable	Group	N	M	SD	F	p
Repurchase	AI	50	5.22	1.44	1.109	0.295
	Marketer	50	5.51	1.34		
Complain	AI	50	4.43	1.80	0.362	0.549
	Marketer	50	4.49	0.83		
Switching	AI	50	4.58	1.79	0.019	0.890
	Marketer	50	4.63	1.83		
Word-to-Mouth	AI	50	5.26	1.31	0.362	0.549
	Marketer	50	5.42	1.35		

Finally, consumer responses to AI pricing versus marketer pricing were analyzed via one-factor ANOVA. The analysis (see Table 4) revealed significance in repurchase (M_AI_ = 2.91 < M_marketer_ = 2.28, *p* = 0.034), word-of-mouth recommendation (M_AI_ = 2.65 < M_marketer_ = 2.00, *p* = 0.007), complaint (M_AI_ = 5.55 < M_marketer_ = 5.99, *p* = 0.034), and switching (M_AI_ = 5.47 < M_marketer_ = 6.00, *p* = 0.034). Thus, in the price difference scenario, AI pricing demonstrated significant impact in all aforementioned aspects (*p* < 0.05), preliminarily verifying the main hypothesis of this study.

**Table 4:** Results of differentiated pricing subject on consumer behavior in scenarios with price differences

Variable	Group	N	M	SD	F	p
Repurchase	AI	50	2.91	1.44	4.646	0.034*
	Marketer	50	2.38	0.98		
Complain	AI	50	5.55	1.15	4.633	0.034*
	Marketer	50	5.99	0.87		
Switching	AI	50	5.47	1.47	5.368	0.023*
	Marketer	50	6.00	0.68		
Word-to-Mouth	AI	50	2.65	1.43	7.494	0.007**
	Marketer	50	2.00	0.89		

^*^p < 0.05

^**^p < 0.01

### Study 1

Study1 was to authenticate the main and mediating effects posited in this research, namely, that AI-initiated pricing, compared to marketer-initiated pricing, elevates consumer repurchase and word-of-mouth behaviors while diminishing complaint behaviors. Additionally, this study aimed to verify the mediating roles of mind perception and ethical perception in these dynamics. Control variables like perceived cost and emotional state were incorporated to mitigate their potential impact on experimental outcomes. This approach aligns with the dual-entitlement theory, which suggests consumer acceptance of price increases when linked to cost rises [4]. Given the substantial investment required for AI implementation, consumers might perceive higher costs associated with AI pricing, influencing their reactions to price differences. Moreover, previous research indicates that emotional responses, particularly negative ones, intensify the adverse impacts of perceived price inequity [34]. In the context of this study, such emotional responses are likely more pronounced towards marketer pricing, potentially skewing experimental results.

#### Experimental design

Study 1 replicated the scenarios from the pre-test for the "with price differences" group and divided participants into two categories: the AI pricing group and the marketer pricing group. In the AI pricing scenario, participants were presented with the following situation: "Please imagine this scenario: HMS Cellular, a top-tier cell phone brand, utilizes pricing robots to determine its product prices. You make a purchase of a cell phone from the HMS store for $4,999, yet you later discover that another customer, Customer A, bought an identical cell phone from the same store on that very day, but for a lower price of $4,799. It's noteworthy that both you and Customer A are regular patrons of HMS. "

For the marketer pricing group, participants were given a different scenario to consider: "Please imagine this scenario: HMS Cellular, a top-tier cell phone brand, utilizes experienced markers to determine its product prices. You make a purchase of a cell phone from the HMS store for $4,999, yet you later discover that another customer, Customer A, bought an identical cell phone from the same store on that very day, but for a lower price of $4,799. It's noteworthy that both you and Customer A are regular patrons of HMS. "

Each participant was required to read the material for 15 s before responding. Participants were asked to imagine themselves in the scenario described in the material and then complete a questionnaire. The questionnaire included independent variables, moderating variables, dependent variables, mediating variables, control variables, screening questions, and demographic variables. The screening questions are designed to prevent random or careless responses by the participants.

#### Data collection and description

Participants from China completed the survey on the Credamo platform (www.credamo.com), where they were randomly assigned to one of the scenarios. For their participation, respondents received a cash payment of $0.5. In this study, All the participants are from China. 284 valid questionnaires were collected, including 92 males and 192 females. The predominant age group was 21–30 years old, comprising 157 individuals or 55.28% of the total; most respondents, 231 in number (81.34%), held a bachelor's degree; a significant portion, 110 people (38.73%), were employed in private enterprises; 116 respondents, accounting for 40.85%, reported spending 4–6 h online daily; and within the past six months, 164 participants (57.74%) spent over 800 yuan monthly on average on shopping.

#### Measurement item

In Study 1, the variables encompassed independent variables, mediating variables (mind perception and ethical perception), dependent variables (repurchase, complaint, switching, and word-of-mouth recommendation), control variables (perceived cost and emotional state), and demographic variables, all consistent with the pre-test. The mind perception, based on Srinivasan & Saria-Abi [52] and Gray et al. [21], included four items, while the ethical perception scale, drawing on Brunk [5], comprised three items. The control variable for perceived cost, referencing Bolton & Alba [4], contained two items, and emotional state, based on Lee & Sternthal [32], included six items. (See Table 1). All items were formulated on a 7-point Likert scale, ranging from 1 ("strongly disagree") to 7 ("strongly agree").

#### Result

The reliability of the variables used in the experiment was assessed. The Cronbach's alpha values for all scales exceeded the critical threshold of 0.7, demonstrating strong reliability, including mind perception(Cronbach's alpha = 0.887), ethical perception (Cronbach's alpha = 0.915), repurchase (Cronbach's alpha = 0.887), complaint (Cronbach's alpha = 0.765), switching (Cronbach's alpha = 0.713), word-of-mouth (Cronbach's alpha = 0.899), and control variables Cost Perception Scale (Cronbachα = 0.873), Perceived Prevalence (Cronbachα = 0.791), and Emotional State (Cronbachα = 0.859). These results indicate that each scale employed in the experiment was reliably constructed.

Then, the perception of the pricing subject was the key manipulated item. Its effectiveness was evaluated through a one-factor analysis of variance. The results showed that (see Table 5): M_AI_ = 6.22 and M_Marketers_ = 1.30, *p* = 0.000. This significant difference between the two groups indicates that the manipulation of the experimental question item was successful.

**Table 5:** Manipulation results of Study 1

Group	N	M	SD	F	p
AI	143	6.22	1.36	1661.79	0.000
Marketer	141	1.30	0.52		

The main effects of this study were evaluated using a one-factor ANOVA, with differentiated pricing doctrine as the independent variable and repurchase, complaint, switching, and word-of-mouth recommendation as the dependent variables. The analysis results, detailed in Table 6, indicated significant differences under the same price difference scenario. Specifically, for repurchase (M_AI_ = 2.87 > M_Marketer_ = 2.43, *p* = 0.004), complaint (M_AI_ = 5.34 < M_Marketer_ = 5.67, *p* = 0.012), switching (M_AI_ = 5.48 < M_Marketer_ = 5.99, *p* = 0.000), and word-of-mouth recommendation (M_AI_ = 2.65 > M_Marketer_ = 2.23, *p* = 0.000), the effects were statistically significant. However, no significant differences were observed in perceived cost (*p* = 0.949) and emotional state (*p* = 0.462) between different pricing subjects. These results suggest that the type of pricing subject employed influences consumer behavior to varying degrees. AI-initiated pricing was found to increase consumer repurchase and word-of-mouth recommendation willingness, while decreasing complaint and switching behaviors, as compared to marketer-initiated pricing. Hence, Hypothesis H1 is supported.

**Table 6:** Results of AI-initiated (marketers-initiated) on consumer behavior

Variable	Group	N	M	SD	F	p
Repurchase	AI	143	2.87	1.37	8.533	0.004**
	Marketer	141	2.43	1.12		
Complain	AI	143	5.34	1.21	6.337	0.012*
	Marketer	141	5.67	0.98		
Switching	AI	143	5.48	1.12	19.918	0.000**
	Marketer	141	5.99	0.79		
Word-to-Mouth	AI	143	2.65	1.28	8.872	0.003**
	Marketer	141	2.23	1.08		
Perceived Cost	AI	143	4.45	1.29	0.004	0.949
	Marketer	141	4.46	1.31		
Emotional State	AI	143	4.62	0.61	0.543	0.462
	Marketer	141	4.68	0.60		

^*^p < 0.05

^**^p < 0.01

The mediating effects were assessed using one-factor ANOVA. The subject of differentiated pricing was designated as the independent variable, while mind perception and ethical perception were the dependent variables. The results, as displayed in Table 7, indicated a significant difference in mind perception between the two experimental groups, with M_AI_ = 3.78 being lower than M_Marketers_ = 5.07 (*p* = 0.000). This finding validates Hypothesis H2. Similarly, a significant difference was observed in ethical perception, with M_AI_ = 3.23 being less than M_Marketers_ = 3.62 (*p* = 0.000), thus supporting Hypothesis H4.

**Table 7:** Results of AI-initiated (marketers-initiated) on mind perception and ethical perception

Variable	Group	N	M	SD	F	p
Mind Perception	AI	143	3.78	1.31	71.271	0.000**
	Marketer	141	5.07	1.27		
Ethical Perception	AI	143	3.23	1.39	14.862	0.000**
	Marketer	141	2.62	1.25		

^**^p < 0.01

In this study, the mediation effect was tested using the PROCESS plug-in in SPSS 22. Model 4 within the Bootstrap method was selected, with a sample size of 5000 and a 95% confidence interval. Dummy variables were designated as independent variables (AI = 1; marketer = 0), with mind perception and ethical perception as mediators. Perceived cost, emotional state, age, and gender were included as control variables. Dependent variables such as repurchase, switching, recommending, and complaining behaviors were analyzed in sequence.

The analysis first examined the mediating roles of mind perception and ethical perception between the differentiated pricing subject and consumer repurchase behavior. Results are presented in Table 8. It was found that the impact of the differentiated pricing subject on consumer repurchase, after accounting for the mediating variables, was not significant (*p* = 0.829 > 0.05). The mediation path "differentiated pricing subject → mind perception → repurchase" was not significant (Bootstrap Lower Level Confidence Interval (LLCI) = -0.072, Bootstrap Upper-Level Confidence Interval (ULCI) = 0.078, including 0). However, the mediation path "differentiated pricing subject → ethical perception → repurchase" displayed a significant mediation effect (Bootstrap LLCI = 0.299, Bootstrap ULCI = 0.757, not including 0), with an effect size of 0.524, indicating a complete mediation.

**Table 8:** Results of parallel mediation tests of mind perception and ethical perception

Consumer Behavior		**Direct effect of X on Y**					
Repurchase	Variable	Effect	SE	LLCI	ULCI	t	P
	Differentiated Pricing subject	-0.028	0.128	-0.280	0.225	-0.216	0.829
		**Indirect effect of X on Y**					
		Effect	Boot SE	Boot LLCI	Boot ULCI	Conclusion	
	Total	0.529	0.128	0.287	0.781		
	Mind Perception(M1)	0.005	0.038	-0.072	0.078	Not Significant	
	Ethical Perception(M2)	0.524	0.119	0.299	0.757	Significant	
Complain		**Direct effect of X on Y**					
	Variable	Effect	SE	LLCI	ULCI	t	P
	Differentiated Pricing subject	0.128	0.131	0.332	-0.131	0.973	0.332
		I**ndirect effect of X on Y**					
		Effect	Boot SE	Boot LLCI	Boot ULCI	Conclusion	
	Total	-0.410	0.106	-0.623	-0.211		
	Mind Perception(M1)	-0.029	0.041	-0.113	0.047	Not Significant	
	Ethical Perception(M2)	-0.0381	0.097	0.166	0.548	Significant	
Switching		**Direct effect of X on Y**					
	Variable	Effect	SE	LLCI	ULCI	t	P
	Differentiated Pricing subject	-0.113	0.115	-0.340	0.114	-0.977	0.329
		**Indirect effect of X on Y**					
		Effect	Boot SE	Boot LLCI	Boot ULCI	Conclusion	
	Total	-0.374	0.962	-0.574	-0.195		
	Mind Perception(M1)	-0.035	0.033	-0.101	0.028	Not Significant	
	Ethical Perception(M2)	-0.340	0.089	-0.524	-0.181	Significant	
Word-of-Mouth		**Direct effect of X on Y**					
	Variable	Effect	SE	LLCI	ULCI	t	P
	Differentiated Pricing subject	-0.083	0.125	-0.328	0.162	-0.667	0.505
		**Indirect effect of X on Y**					
		Effect	Boot SE	Boot LLCI	Boot ULCI	Conclusion	
	Total	0.534	0.121	0.293	0.770		
	Mind Perception(M1)	0.041	0.036	-0.027	0.117	Not Significant	
	Ethical Perception(M2)	0.492	0.114	0.271	0.711	Significant	

In the second phase of analysis, the study investigated the mediating roles of mind perception and ethical perception between differentiated pricing subjects and consumer complaints. The results, as outlined in Table 8, indicated that after including the mediating variables, the effect of the differentiated pricing subject on consumer complaints was not statistically significant (*p* = 0.332 > 0.05, Lower-Level Confidence Interval (LLCI) = -0.332, Upper-Level Confidence Interval (ULCI) = -0.131). The mediation path "differentiated pricing subject → mind perception → complaints" was found to be non-significant (Bootstrap LLCI = -0.131), and the mediation path "differentiated pricing subject → ethical perception → complaints" was also not significant (Bootstrap LLCI = -0.113, Bootstrap ULCI = 0.047, including 0). However, in the scenario of "differentiated pricing subject → ethical perception → complaints," a significant mediation effect was observed (Bootstrap LLCI = 0.166, Bootstrap ULCI = 0.548, not including 0), with an effect size of -0.0381, indicating complete mediation.

Then, it evaluated the mediating roles of mind perception and ethical perception between the differentiated pricing subject and consumer switching behavior. The results, presented in Table 8, indicated that after incorporating the mediating variables, the influence of the differentiated pricing subject on consumer switching was not statistically significant (*p* = 0.329 > 0.05, Lower Level Confidence Interval (LLCI) = -0.340, Upper-Level Confidence Interval (ULCI) = 0.114). The mediation path "differentiated pricing subject → mind perception → switching" was found to be non-significant (Bootstrap LLCI = -0.101, Bootstrap ULCI = 0.028, including 0). Likewise, the mediation path "differentiated pricing subject → ethical perception → switching" was also not significant (Bootstrap LLCI = -0.101, Bootstrap ULCI = 0.028, including 0). However, the path "differentiated pricing subject → ethical perception → switching" demonstrated a significant mediating effect (Bootstrap LLCI = -0.524, Bootstrap ULCI = -0.181, not including 0), with an effect size of -0.340, indicating complete mediation.

Finally, the analysis of the mediating roles of mind perception and ethical perception between different pricing subjects and word-of-mouth recommendation is presented in Table 8. The results indicate no significant effect of the differentiated pricing subject on word-of-mouth recommendation after incorporating the mediating variables (*p* = 0.505 > 0.05, LLCI = -0.328, ULCI = 0.162). Furthermore, the mediation path "differentiated pricing subject → mind perception → word-of-mouth recommendation" is not significant (Bootstrap LLCI = -0.027, Bootstrap ULCI = 0.117, including 0). However, the path "differentiated pricing subject → ethical perception → word-of-mouth recommendation" demonstrates a significant mediation effect (Bootstrap LLCI = 0.271, Bootstrap ULCI = 0.711, not including 0), signifying a complete mediation.

Integrating the outcomes of the mediation analysis, it becomes evident that ethical perception acts as a mediator in the relationship between differentiated pricing subjects and various aspects of consumer behavior, including repurchase, complaint, switching, and word-of-mouth recommendation. Consequently, this supports hypothesis 5. On the contrary, mind perception does not exhibit a significant mediating effect on the impact of differentiated pricing subjects on these consumer behaviors, so H3 is not supported.

#### Discuss

Study 1 incorporated two experimental groups: AI pricing and marketer pricing groups. These groups were subjected to a scenario-imagination manipulation to immerse subjects in specific pricing contexts. The primary aim was to assess the influence of different differentiated pricing strategies on consumer behavior. The findings indicated that AI-initiated pricing, as opposed to marketer-initiated pricing, led to increased repurchase and word-of-mouth recommendation behaviors and reduced complaint and switching behaviors, thereby validating Hypothesis H1. Additionally, a one-factor ANOVA revealed that consumers' mind perception of AI was lower compared to that of marketers, while their ethical perception was higher, confirming Hypotheses H2 and H4. Building on these insights, the study further examined the mediating roles of mind perception and ethical complete mediator, affirming Hypothesis H5. In contrast, mind perception did not demonstrate a significant mediating effect, leading to the non-confirmation of Hypothesis H3.

The significant disparity in consumers' mind perceptions of AI and marketers, despite the non-significant role of the mind as a mediator, may be attributed to two key factors: Firstly, scholars such as Zhao et al. [66] and Hayes [24] suggest that when an independent variable impacts a dependent variable through multiple mediating variables, these mediating effects can negate each other, particularly if they operate in opposing directions. In this study, the contrasting influences of mind perception and ethical perception on the dependent variable might lead to the non-significant mediation effect of mind perception due to their antagonistic actions. Secondly, the inconsistency between the experimental results and the hypotheses could stem from a lack of personal experience with differentiated pricing among some participants. This gap in experience might result in an incomplete understanding of current AI pricing strategies, thereby influencing their responses and perceptions in the experiment.

### Study 2

Study 2 aimed to investigate the moderating effects of perceived enterprise control on the main effects, mind perception, and ethical perception. The study posited that the type of firm ownership over service providers significantly influences consumers' perceptions of control. For instance, consumers tend to perceive greater enterprise control and attribute more dissatisfaction with service outcomes when they are informed that a service provider is a regular employee of the firm, rather than an outsourced worker. Given the diverse ownership forms of AI systems, including independently developed and implemented systems like IBM's and Jingdong's "Y-SMART SC" smart pricing, as well as Yousin's used-car platform, and third-party AI systems such as those used by the majority of eBay merchants from SLD, this variability offers a rich context for analysis. In terms of manipulating perceived enterprise control, the study classified enterprise self-developed AI systems and formally trained enterprise pricing employees as indicators of high perceived enterprise control. Conversely, AI systems not developed by the enterprise and non-formal employees were considered as low perceived control.

#### Experimental design

Study 2 employed a between-group design, encompassing differentiated pricing subjects (AI vs. marketer) and perceived firm control (high vs. low). Participants were randomly assigned to one of these experimental groups. The manipulation was conducted using a scenario-imagery method. The experiment's setting was situated in the C2C (Consumer-to-Consumer) domain, specifically within a second-hand trading platform where prices are evaluated either by AI or by marketers. For manipulating perceived enterprise control, the experiment drew upon the research of Song and He [51]. The specific experimental scenarios, designed to reflect varying levels of perceived firm control, were as follows:

- AI × Low Perceived Firm Control Group: Please imagine this scenario: “The HMS Platform operates as a marketplace for second-hand trading. You have recently decided to sell an old cell phone that you no longer use. The platform's AI pricing robot, purchased from another company and was not developed and designed in-house, appraises your phone at $1,400. However, you discover that Customer A, who is offering a cell phone identical to yours in terms of age, condition, and model, has had their phone valued at $1,500 by the same robot."
- Marketers × Low Perceived Firm Control Group: Please imagine this scenario: “The HMS Platform operates as a marketplace for second-hand trading. You have recently decided to sell an old cell phone that you no longer use. The platform's marketer, who is an outsourced employee rather than a regular staff member of HMS, appraises your phone at $1,400. However, you discover that Customer A, who is offering a cell phone identical to yours in terms of age, condition, and model, has had their phone valued at $1,500 by the same marketer."
- AI × High Perceived Firm Control Group: Please imagine this scenario: “The HMS Platform operates as a marketplace for second-hand trading. You have recently decided to sell an old cell phone that you no longer use. The platform's AI pricing robot, which was developed and designed in-house and follows the set pricing rules by HMS, appraises your phone at $1,400. However, you discover that Customer A, who is offering a cell phone identical to yours in terms of age, condition, and model, has had their phone valued at $1,500 by the same robot."
- Marketers × High Perceived Firm Control Group: Please imagine this scenario: “The HMS Platform operates as a marketplace for second-hand trading. You have recently decided to sell an old cell phone that you no longer use. The platform's marketer, who has undergone specialized pricing training provided by HMS and has successfully passed the associated assessment, appraises your phone at $1,400. However, you discover that Customer A, who is offering a cell phone identical to yours in terms of age, condition, and model, has had their phone valued at $1,500 by the same marketer."

Each participant was required to read the material for 15 s before responding. Participants were asked to imagine themselves in the scenario described in the material and then complete a questionnaire. The questionnaire included independent variables, moderating variables, dependent variables, mediating variables, control variables, screening questions, and demographic variables. The screening questions are designed to prevent random or careless responses by the participants.

#### Data collection and description

Participants from China completed the survey on the Credamo platform (www.credamo.com), where they were randomly assigned to one of the scenarios. For their participation, respondents received a cash payment of $ 0.5. In this study, All the participants are from China. 357 valid questionnaires were collected. The distribution of participants across the experimental groups was as follows: 92 individuals were in the "AI × Low Perceived Enterprise Control" group, 97 in the "Marketers × Low Perceived Enterprise Control" group, 84 in the "AI × High Perceived Enterprise Control" group, and 84 in the "Marketers × High Perceived Enterprise Control" group. The demographic breakdown of the respondents was as follows: 164 males and 193 females participated; the predominant age group was 21–30 years old, comprising 170 individuals or 47.6% of the total; most respondents, 260 in number (72.8%), held a bachelor's degree; a significant portion, 166 people (46.6%), were employed in private enterprises; 140 respondents, accounting for 39.2%, reported spending 4–6 h online daily; and within the past six months, 209 participants (58.5%) spent over 800 yuan monthly on average on shopping.

#### Measurement item

In study 2, the variables encompassed independent variables, mediating variables (mind perception and ethical perception), dependent variables (repurchase, complaint, switching, and word-of-mouth recommendation), control variables (perceived cost and emotional state), and demographic variables, all consistent with the study 1. Perceived enterprise control was adapted from the research of Song and He [51], shown in Table 1. All items were formulated on a 7-point Likert scale, ranging from 1 ("strongly disagree") to 7 ("strongly agree").

#### Result

The Cronbach's alpha values for all scales exceeded the critical threshold of 0.8, demonstrating strong reliability, including mind perception(Cronbach's alpha = 0.934), ethical perception (Cronbach's alpha = 0.903), repurchase (Cronbach's alpha = 0.945), complaint (Cronbach's alpha = 0.827), switching (Cronbach's alpha = 0.858), word-of-mouth (Cronbach's alpha = 0.935), and control variables Cost Perception Scale (Cronbachα = 0.856), and Emotional State (Cronbachα = 0.841). These results indicate that each scale employed in the experiment was reliably constructed.

In study 2, the efficacy of the pricing subject manipulation was evaluated using a two-factor ANOVA, with the pricing subject as the dependent variable. The results of this analysis are presented in Table 9. The mean score for the Artificial Intelligence group was M_AI_ = 6.10, while for the Marketers group, it was M_Marketers_ = 1.47. The substantial difference between these scores, with a *p*-value of 0.000, is statistically significant. This significant disparity confirms the successful manipulation of the pricing subject's question item. Then, the study proceeded to test the manipulation of perceived enterprise control, employing a two-factor ANOVA with perceived enterprise control as the dependent variable. The outcomes of this test are detailed in Table 10. The results indicated a mean score of M_Low PCC_ = 4.60 and M_High PCC_ = 5.56. The marked difference between these two scores, evidenced by a *p*-value of 0.000, is statistically significant. This significant disparity confirms the successful manipulation of perceived enterprise control.

**Table 9:** Manipulation results of differentiated pricing subject

Group	N	M	SD	F	p
AI	176	6.10	1.24	1765.44	0.000
Marketer	181	1.47	0.81

**Table 10:** Manipulation results of perceived enterprise control

Group	N	M	SD	F	p
Low Perceived Enterprise Control	189	4.60	1.59	40.75	0.000
High Perceived Enterprise Control	168	5.56	1.18		

Firstly, the moderating role of perceived enterprise control on the impact of differentiated pricing subjects (AI vs. marketers) on various consumer behaviors was investigated. To this end, the two categorical variables were recorded: differentiated pricing subject was set as AI = 1 and marketers = 0, and perceived enterprise control as low = 0 and high = 1. These variables served as independent variables in a two-factor analysis, with repurchase, complaint, switching, word-of-mouth recommendation, mind perception, and ethical perception as dependent variables. The results, presented in Table 11, revealed that perceived enterprise control significantly moderated the relationship between differentiated pricing subjects and consumer behaviors such as repurchase (*p* < 0.05), complaint (*p* < 0.05), switching (*p* < 0.05), and word-of-mouth recommendation (*p* < 0.05). Additionally, perceived enterprise control also played a moderating role in the effects of differentiated pricing subjects on mind perception (*p* < 0.05) and ethical perception (*p* < 0.05).

**Table 11:** Results of the moderating perceived enterprise control

Source	Variable	*R*^2^	DF	MS	F	p
Pricing Subject × Perceived Enterprise Control	Repurchase	15.151	1	15.151	8.543	0.004**
	Complain	20.114	1	20.114	19.639	0.000**
	Switching	54.325	1	54.325	44.809	0.000**
	Word-of-Mouth	43.495	1	43.495	33.676	0.000**
	Mind Perception	138.057	1	138.057	87.448	0.000**
	Ethical Perception	72.629	1	72.629	55.986	0.000**

^**^p < 0.01

Perceived firm control was divided into two groups: low perceived firm control and high perceived firm control. Within the low perceived firm control group (*N* = 189), the AI group comprised 92 subjects, while the marketer group included 97 subjects. For the high perceived firm control group (*N* = 168), the AI group had 84 subjects, and the marketer group had 83 subjects. Using the subject of differentiated pricing as the independent variable, repurchase, complaint, switching, word-of-mouth recommendation, mind perception, and ethical perception were established as the dependent variables for an analysis of variance. The results, as detailed in Table 12, indicate that in the low perceived control group, differentiated pricing subjects significantly influenced repurchase (M_AI_ = 3.576 > M_marketer_ = 3.110, *p* = 0.0049), complaint (M_AI_ = 5.152 < M_marketer_ = 5.618, *p* = 0.007), switching (M__AI_ = 5.239 < M_marketer_ = 5.964, *p* = 0.000), and word-of-mouth recommendation (M_AI_ = 3.192 > M_marketer_ = 2.316, *p* = 0.000). Similarly, mind perception (M_AI_ = 3.182 < M_marketer_ = 5.196, *p* = 0.000) and ethical perception (M_AI_ = 3.547 < M_marketer_ = 2.584, *p* = 0.000) also showed significant results. These findings provided further validation for Hypotheses 1, 2, and 3.

**Table 12:** Results of pricing subject on consumers under high and low perceived enterprise control groups

Group	Variables	Source	M	SD	F	p
High Perceived Enterprise Control	Repurchase Complain	AI	3.576	1.702	3.935	0.049
		Marketer	3.110	1.537		
	Switching Word-of-Mouth	AI	5.152	1.285	7.532	0.007
		Marketer	5.618	1.044		
	Mind Perception	AI	5.239	1.482	17.815	0.000**
		Marketer	5.964	0.795		
	Repurchase Complain	AI	3.192	1.615	20.955	0.000**
		Marketer	2.316	0.946		
	Switching Word-of-Mouth	AI	3.182	1.437	132.601	0.000**
		Marketer	5.196	0.924		
	Mind Perception	AI	3.547	1.699	24.355	0.000**
		Marketer	2.584	0.874		
Low Perceived Enterprise Control	Repurchase Complain	AI	2.675	1.093	7.482	0.007
		Marketer	3.169	1.238		
	Switching Word-of-Mouth	AI	6.016	0.633	13.266	0.000**
		Marketer	5.562	0.947		
	Mind Perception	AI	6.042	0.582	32.084	0.000**
		Marketer	5.151	1.318		
	Repurchase Complain	AI	2.262	0.957	15.104	0.000**
		Marketer	2.888	1.118		
	Switching Word-of-Mouth	AI	5.155	1.208	3.745	0.055
		Marketer	4.741	1.537		
	Mind Perception	AI	2.587	0.958	40.222	0.000**
		Marketer	3.514	0.930		

^**^p < 0.01

In the high perceived control group, the impact of differentiated pricing subjects on consumer behaviors and perceptions showed significant outcomes. Specifically, repurchase was higher for the marketer group (M_AI_ = 2.675 < M_marketer_ = 3.169, *p* = 0.007), while complain (M_AI_ = 6.016 > M_marketer_ = 5.562, *p* = 0.000), switch (M_AI_ = 6.042 > M_marketer_ = 5.151, *p* = 0.000), and word-of-mouth recommendation (M_AI_ = 2.262 < M_marketer_ = 2.888, *p* = 0.000) were significantly affected. Additionally, ethical perception also showed a significant difference (M_AI_ = 2.587 < M_marketer_ = 3.514, *p* = 0.000). These results, in combination with those from the low perceived control group, validate Hypotheses H6a and H6c. However, the effect on mind perception was not significant (M_AI_ = 5.155 > M_marketer_ = 4.741, *p* = 0.000), which aligns with Hypothesis H6b.

In the final analysis, Model 8 of the PROCESS plug-in in SPSS was employed to examine the mediating roles of mind perception and ethical perception, considering their modifiability. The dependent variables used were repurchase, complaint, switching, and word-of-mouth recommendation. The independent variable was the pricing subject (AI = 1, marketer = 0), while perceived enterprise control (low = 0, high = 1) was analyzed as a moderating variable. Perceived cost and emotional state were included as control variables. The results are depicted in Table 13. When repurchase, complaint, switching, and word-of-mouth recommendation served as dependent variables, none of the 95% confidence intervals for ethical perception included 0, and the index for the moderated mediating role likewise excluded 0. This indicates that the moderated mediating role of ethical perception is sustained. In cases where the dependent variables were repurchase and switching, the 95% confidence intervals for mind perception did not contain 0, and the moderated effect index also excluded 0, partially establishing the moderated mediation role of mind perception. However, for complaint and word-of-mouth recommendation, the 95% confidence intervals for mind perception included 0, as did the moderated effect index, suggesting that the moderated mediation role of mind perception is only partially supported.

**Table 13:** Analysis of moderating effects

Path: AI-Mind Perceived /Ethical Perceived-Repurchase				
Mediators	Level	Effect	SE	95% Bootstrap CI
Mind Perception	Low	-0.2339	0.860	[-0.4030, -0.0682]
	High	0.0429	0.0310	[-0.0041,0.1156]
Index		0.2768	0.1061	[0.0761,0.4969]
Ethical Perception	Low	0.5631	0.1323	[0.3070,0.8310]
	High	-0.6850	0.1080	[-0.8990, -0.4748]
Index		-1.2481	0.1764	[-1.5945, -0.9101]
Path: AI-Mind Perceived /Ethical Perceived-Complain				
Mediators	Level	Effect	SE	95% Bootstrap CI
Mind Perception	Low	0.1024	0.0826	[-0.0657,0.2647]
	High	-0.0188	0.0212	[-0.0735,0.0104]
Index		-0.1211	0.999	[-0.3256,0.0741]
Ethical Perception	Low	-0.3019	0.0755	[-0.4600, -0.1634]
	High	0.3672	0.0748	[-0.2300,0.5276]
Index		0.6691	0.1214	[0.4473,0.9239]
Path: AI-Mind Perceived /Ethical Perceived- Switching				
Mediators	Level	Effect	SE	95% Bootstrap CI
Mind Perception	Low	0.3139	0.1019	[0.1098,0.5116]
	High	-0.0575	0.0378	[-0.1421,0.058]
Index		-0.3714	0.1225	[-0.6177, -0.1300]
Ethical Perception	Low	-0.2496	0.0698	[-0.3988, -0.1271]
	High	0.3036	0.0669	[0.1826, 0.4441]
Index		0.5532	0.1161	[0.3475, 0.7897]
Path: AI-Mind Perceived /Ethical Perceived- Word-of-Mouth)				
Mediators	Level	Effect	SE	95% Bootstrap CI
Mind Perception	Low	-0.1425	0.0892	[-0.3192,0.303]
	High	0.0261	0.0245	[-0.0056,0.0853]
Index		0.1686	0.1083	[-0.0341,0.3898]
Ethical Perception	Low	0.3996	0.1010	[0.2116,0.6076]
	High	-0.4861	0.0874	[-0.6679,0.3264]
Index		-0.8857	0.1522	[-1.2040, -0.6107]

#### Discuss

Study 2 implemented a between-groups design involving 2 differentiated pricing subjects (AI vs. marketers) × 2 levels of perceived enterprise control (low vs. high), using a scenario-imagery manipulation method. Through two-factor ANOVA, the study examined the moderating role of perceived enterprise control in the context of differentiated pricing subjects on consumer behavior, mind perception, and ethical perception. These findings indicated that under conditions of low consumer-perceived enterprise control, AI-initiated pricing (vs. marketer) resulted in higher repurchase and word-of-mouth recommendation behaviors, fewer complaints, and switching behaviors. Additionally, consumers had lower mind perceptions and higher ethical perceptions of AI (vs. marketers), reinforcing Hypotheses 1, 2, and 4. Conversely, when consumer-perceived enterprise control was high, the effects of AI pricing (vs. marketers) on consumers were reversed. In this scenario, consumers exhibited more negative responses to AI pricing, including lower repurchase and word-of-mouth recommendation behaviors, increased complaints, and switching behaviors. Furthermore, consumers' mind perceptions of AI (vs. marketers) were higher, while ethical perceptions were lower. Thus, the moderating effect of perceived enterprise control was validated, affirming Hypotheses H6a, H6b, and H6c.

Additionally, study 2 assessed the moderated mediation of mind and ethical perceptions. The moderated mediation of ethical perceptions was established, while the moderated mediation of mind perceptions was confirmed in the contexts of repurchase and switching behaviors but not in complaining and word-of-mouth recommendation contexts. This resulted in a partial establishment of the moderated mediation role of mind perceptions.

## Conclusions

This study, grounded in an ethical and ethical perspective and drawing upon attribution theory and S–O-R theory, focused on mind perception and ethical perception as mediating variables and perceived enterprise control as a moderating variable. A total of three experiments were conducted, yielding 841 valid questionnaires. The research findings indicate that, when confronted with price differences, consumers who are at a price disadvantage exhibit more tolerance towards AI-initiated pricing (vs. marketers), as evidenced by higher repurchase and word-of-mouth recommendation behaviors, and fewer complaints and switching behaviors. Perceived enterprise control was found to play a significant moderating role, such that when these consumers perceive that corporations can manipulate AI-initiated pricing, AI-initiated pricing (vs. marketers) tends to evoke more negative consumer behavior, and may even backfire against marketers.

The insights from this paper can offer valuable marketing guidance to firms and platforms either currently utilizing or considering the adoption of AI pricing strategies. However, it's important to note that the study primarily considered the behavioral responses of price-disadvantaged consumers. Although pre-test results indicated no significant difference in behavioral responses when there was no price difference between AI-initiated pricing and marketer pricing, the psychological and behavioral reactions of consumers who benefit from price advantages under AI-initiated pricing and marketer pricing might differ. Recent studies have suggested that even when consumers gain a price advantage, they may still perceive personalized pricing as unfair due to feelings of guilt, leading to certain negative behaviors.

Therefore, future research could expand the scope to include consumers who gain price advantages, analyzing their responses in comparison with price-disadvantaged consumers. Such comprehensive research could provide deeper insights into the broader implications of AI-initiated pricing and marketer-initiated pricing strategies across different consumer segments.

## Significance

The study theoretically demonstrates that AI and marketer-differentiated pricing variably influence consumer’s repurchase intentions, word-of-mouth recommendations, complaints, and switching behaviors. This enriches human-AI comparison research within the marketing context, expands algorithmic pricing studies, and delves deeper into the price-sourcing effect. Additionally, it contributes to research on mind and ethical perception in pricing. Unlike previous studies focused on price fairness perception, this research adopts an ethical and ethical perspective. It investigates the mediating role of pricing subjects (AI vs. marketers) on consumer behavior through micro-personal mind perception and macro-social ethical perception. This approach unveils another psychological pathway for consumers facing price differences, elucidating AI-initiated pricing's impact on consumer psychology and behavior, thereby broadening research on mind and ethical perceptions in pricing.

Furthermore, the study examines the boundaries of perceived enterprise control's effect on consumer behavior in differentiated pricing contexts (AI vs. marketers), revealing ethical dilemmas for firms and platforms using AI-initiated pricing. While AI-initiated pricing can boost profits and create consumer surplus, perceived manipulation by firms significantly increases consumer complaints and switching behaviors, while drastically reducing repurchase intentions and word-of-mouth recommendations.

Practically, the findings aid companies and platforms in understanding consumers' psychological reactions and behavioral responses to AI-initiated pricing, offering insights for marketing communication or service remediation. Despite AI pricing's potential for profit and consumer surplus, differentiated pricing can still trigger negative consumer behaviors. A comparison with marketer-differentiated pricing shows a higher consumer tolerance for A-initiated pricing, evidenced by increased repurchase and recommendation behaviors and decreased complaints and switching behaviors.

Moreover, the research advises companies and platforms that AI-initiated pricing is not a universally applicable solution. Discovering enterprise capabilities to manipulate AI-initiated pricing prompts consumer behaviors as negative as, or worse than, those elicited by marketer-differentiated pricing. Hence, this study helps businesses and platforms grasp the dual aspects of AI-initiated pricing to develop reasonable pricing strategies.

## Limitation

Most of the research hypotheses in this paper have been validated, but there are still shortcomings and limitations.

First, the research methodology includes pre-experiments and two formal experiments based on scenario experiments. Although the variables were effectively manipulated and the hypotheses supported, these methods may lack external validity. Future research should extend to more realistic environments (e.g., field experiments) to increase authenticity by simulating real-life scenarios. Alternatively, Python technology could be used to capture negative behavioral data of price-sensitive consumers on e-commerce platforms, enhancing the study's external validity and making it more applicable to management practice.

Secondly, this study only considers the behavioral responses of price-disadvantaged consumers, but there are also price-advantaged consumers and those who pay the same price. The pre-experiment showed no significant difference in the behavioral responses of consumers when there was no price difference between AI pricing and marketers' pricing. However, would the psychology and behavior of consumers with a price advantage differ between AI pricing and marketer pricing? Therefore, future research could include price-advantaged consumers and compare their responses to those of price-disadvantaged consumers.

## Implication

We would strongly suggest future researchers to use Online Photovoice (OPV) [15, 54], Online Interpretative Phenomenological Analysis (OIPA), and Community-Based Participatory Research (CBPR) [12, 13] to conduct research on the same or similar topics, which gains deeper insights into consumer behavior. These innovative methods can act with and for people dealing with this subject for more grounded research to capture the thoughts, feelings, images, and behaviors of people from their own unique experiences to set the ground for more effective services.

Besides, future researchers can use qualitative or mixed methods to explore OPV. And educators/trainers etc. also can use OPV for experiential activities to increase group and organizational synergy. OPV and OIPA allows one to use one of the most simple and straight approach to analyze the data in one of the most comprehensive and meaningful way.

## Data Availability

No datasets were generated or analysed during the current study.
